# Early Blood Gas Predictors of Bronchopulmonary Dysplasia in Extremely Low Gestational Age Newborns

**DOI:** 10.1155/2014/210218

**Published:** 2014-05-15

**Authors:** Sudhir Sriram, Joy Condie, Michael D. Schreiber, Daniel G. Batton, Bhavesh Shah, Carl Bose, Matthew Laughon, Linda J. Van Marter, Elizabeth N. Allred, Alan Leviton

**Affiliations:** ^1^Department of Pediatrics, University of Chicago, 5841 South Maryland Avenue MC 6060, Chicago, IL 60637, USA; ^2^Department of Pediatrics, Southern Illinois School of Medicine, 301 North 8th Street, Springfield, IL 62794, USA; ^3^Department of Pediatrics, Bay State Medical Center, 759 Chestnut Street, Springfield, MA 01199, USA; ^4^Department of Pediatrics, University of North Carolina, 101 Manning Drive, Chapel Hill, NC 27599, USA; ^5^Department of Pediatrics, Harvard Medical School, 220 Longwood Drive, Boston, MA 02115, USA; ^6^Division of Newborn Medicine, Children's Hospital, 300 Longwood Avenue, Boston, MA 02115, USA; ^7^Division of Newborn Medicine, Brigham and Women's Hospital, 75 Francis Street, Boston, MA 02115, USA; ^8^Department of Neurology, Harvard Medical School, 220 Longwood Drive, Boston, MA 02115, USA; ^9^Department of Biostatistics, Harvard School of Public Health, 655 Huntington Avenue, Boston, MA 02115, USA; ^10^Department of Neurology, Children's Hospital, 300 Longwood Avenue, Boston, MA 02115, USA

## Abstract

*Aim*. To determine among infants born before the 28th week of gestation to what extent blood gas abnormalities during the first three postnatal days provide information about the risk of bronchopulmonary dysplasia (BPD). *Methods*. We studied the association of extreme quartiles of blood gas measurements (hypoxemia, hyperoxemia, hypocapnea, and hypercapnea) in the first three postnatal days, with bronchopulmonary dysplasia, among 906 newborns, using multivariable models adjusting for potential confounders. We approximated NIH criteria by classifying severity of BPD on the basis of the receipt of any O_2_ on postnatal day 28 and at 36 weeks PMA and assisted ventilation. *Results*. In models that did not adjust for ventilation, hypoxemia was associated with increased risk of severe BPD and very severe BPD, while infants who had hypercapnea were at increased risk of very severe BPD only. In contrast, infants who had hypocapnea were at reduced risk of severe BPD. Including ventilation for 14 or more days eliminated the associations with hypoxemia and with hypercapnea and made the decreased risk of very severe BPD statistically significant. *Conclusions*. Among ELGANs, recurrent/persistent blood gas abnormalities in the first three postnatal days convey information about the risk of severe and very severe BPD.

## 1. Introduction

Despite the improved survival of extremely low gestational age newborns (ELGANs) defined as infants born at <28 weeks' gestation, bronchopulmonary dysplasia (BPD) remains prevalent and an important healthcare burden to patients, their families, and society [1–4]. Identifying early, perhaps remediable, indicators of later pulmonary disorders has the potential to contribute to the reduction of the risk of these disorders.

Blood gas abnormalities and acid-base disturbances can occur as a result of lung immaturity that can be complicated by comorbidities, such as perinatal infection or pulmonary hypoplasia [5–10]. Likewise, medical management, including permissive hypercapnea, also can predispose, intentionally or unintentionally, to blood gas abnormalities [11–13].

In the presurfactant era, low PCO_2_ levels were associated with increased risk, and high PCO_2_ levels were associated with decreased risk of BPD [15–17]. In the postsurfactant era, BPD has been predicted by oxygen requirement, abnormal chest X-ray, and ventilator dependency at 4 days of age [18]. The relationship between early blood gas abnormalities and the development of BPD has not been well studied since the wide-spread acceptance of antenatal steroids, surfactant replacement therapy, and permissive hypercapnea.

The objective of the present study was to examine the relationship between blood gas abnormalities in the first three postnatal days and BPD risk.

## 2. Subjects and Methods

The ELGAN study was originally designed to detect structural and functional neurological abnormalities in infants born before 28 weeks of gestational age at 14 participating centers in the USA during the years 2002–2004 [19]. Approval was obtained from the institutional review board (IRB) at each participating institution. Consent was obtained from mothers, either antenatally or after delivery, based on the human research committee recommendations and/or individual clinical situation. Of 1506 infants enrolled, information on placental microbiology, blood gases, and BPD diagnosis was available for 906 infants.

The estimates of gestational age (GA) were based on availability of information to best determine the most accurate gestational age. The most accurate GA estimates were obtained from dates of embryo retrieval, intrauterine insemination, or by fetal ultrasound prior to 14 weeks (62%). The next reliable GA estimate was given to fetal ultrasound at greater than 14 weeks (29%), LMP (7%), and postnatal estimation of gestational age, as recorded in the neonatal intensive care unit log book (1%). The birth weight *Z*-score reflects the number of standard deviations the infant's birth weight is above or below the median weight of infants of the same gestational age in a standard data set [14].

We collected all the physiology, laboratory, and therapy data for the first 12 hours needed to calculate a score for neonatal acute physiology (SNAP-II) and a score for neonatal acute physiology perinatal extension (SNAPPE-II) [20].

We classified infants by the number of days they received mechanical ventilation (either high-frequency or conventional mechanical ventilation) from birth to 36 weeks' postmenstrual age (PMA). For the analyses presented here, we limited our analyses to the trichotomy of <7 days, 7–13 days, and 14 or more days of ventilation.

### 2.1. Blood Gas Collection and Classification

PO_2_ and PCO_2_ were measured almost routinely on the first three postnatal days and we collected the lowest, modal, and highest values on each day [21, 22]. The first postnatal day was defined as the first 24 hours after delivery plus the additional hours until midnight. Each successive day then began and ended at midnight [2]. The median number of blood gases obtained on each day declined rapidly from 8 on day 1 to 4 on day 3. With the exception of 7 ELGANs on day 1, 15 ELGANs on day 2, and 60 ELGANs on day 3 who had venous/capillary measurements, all other values were from arterial specimens.

ELGANs were classified by gestational age (23-24, 25-26, and 27 weeks) and blood specimens were classified by the postnatal day they were obtained (1, 2, 3). Quartiles of PaO_2_ and PCO_2_ were calculated for each of 9 gestational age and postnatal day groups, separately for arterial and venous values (i.e., 18 groups), and blood gases of each infant were assigned to his/her quartile based on membership in one of these 18 groups. We considered an infant to be exposed to abnormal blood gas values, if she/he had a measurement in the lowest quartile and, separately, the highest quartile on at least two of three postnatal days. Values in the lowest or highest quartile on just one postnatal day were not considered an exposure.

Because we collected the minimum and maximum blood gas values each day, we cannot tell if the values of PCO_2_ and/or PO_2_ are from the same specimen. Since no protocol was established for sampling blood gases, all measurements were obtained at the discretion of the clinical team caring for each infant.

### 2.2. Modified Definitions of BPD

Because we did not collect information about percentage of oxygen delivered on postnatal day 28 or at 36 weeks PMA, we could not use NIH criteria for BPD [23, 24].

We approximated the NIH criteria as best as we could by replacing <30% and >30% with any oxygen. Thus, mild and moderate BPD are defined as a need for supplemental oxygen ≥28 days, but not at 36 weeks PMA, while severe BPD is defined as a need for supplemental oxygen at 36 weeks PMA, but not requiring ventilation assistance, and a classification of very severe BPD required a need for both supplemental oxygen and ventilation assistance at 36 weeks PMA.

### 2.3. Placenta

Delivered placentas were placed in a sterile exam basin and transported to a sampling room, where they were biopsied under sterile conditions. Eighty-two percent of the samples were obtained within 1 hour of delivery. The microbiologic and histologic procedures are described in detail elsewhere [25, 26]. Briefly, inflammation was defined as any of the following four lesions: (a) chorionic plate inflammation of grade 3 (neutrophils up to amnionic epithelium) and stage 3 (>20 neutrophils/20x), (b) external membrane inflammation of grade 3 (numerous large or confluent foci of neutrophils), (c) umbilical cord inflammation of grade 3 or higher (neutrophils in perivascular Wharton's jelly), and (d) neutrophilic infiltration into fetal stem vessels in the chorionic plate.

### 2.4. Statistical Analysis

We evaluated the hypothesis that ELGANs who have a blood gas value in an extreme quartile on at least 2 of the first 3 postnatal days are at increased risk for developing BPD. Four extremes in blood gases were considered: lowest and highest quartiles of PO_2_ and PCO_2_. Because only 80 of the 906 infants in our sample did not have any BPD by our minor modification of NIH criteria, having this group of infants serve as the referent group for multivariable analyses prominently limited statistical power. Consequently, we decided to group these infants who were not oxygen dependent on day 28 with those who were not dependent at 36 weeks PMA (*N* = 314) to form the referent group for all multivariable analyses of BPD risk.

We selected variables as confounders if in our data they were associated with both a blood gas extreme and with one of the pulmonary disorders with probabilities ≤0.25 [27]. After reviewing a broad range of potential confounders, we found that six variables were associated with both a blood gas extreme and severe or very severe BPD, including conception assistance, maternal fever during pregnancy, relative fetal growth restriction (defined as a birth weight more than one standard deviation below the expected median), recovery of* Mycoplasma* from the placenta, and illness severity indicators (SNAP-II and SNAPPE-II). The number of days ventilated was also associated with both blood gas disturbances and BPD, but we elected not to include this variable in the top part of Table 4 because it might have been an intervening variable between the blood gas abnormality and BPD. Rather, we added this potential confounder to the bottom part of Table 4. Comparison of the top to the bottom part of Table 4 allows an appreciation of how much the duration of ventilation variable might be an intervening variable between each blood gas disturbance and BPD.

All models included a hospital cluster term to account for the likelihood that infants born at one hospital are more alike and more likely to have received the same respiratory care than those born at another hospital [28]. The contributions of blood gas abnormalities to severity of BPD are given as odds ratios with 95% confidence intervals, controlling for potential confounders. In an effort to balance the risks of type 1 and type 2 errors, while evaluating only 4 highly related blood gas extremes, we chose to describe the precision of odds ratio estimates with 95% confidence intervals.

## 3. Results

Of the 1506 infants enrolled in the ELGAN Study, 1172 had blood gas assessments and 906 survived until 36 weeks PMA and had information about all variables in the multivariable models. Infants who did not have a blood gas measurement on all 3 of the first 3 postnatal days were less likely than others to have a high SNAPPE-II, lower mean arterial pressures, and to have had day-2 blood gas measurements that were in an extreme quartile. Fully 29% had a SNAPPE-II ≥45.

The values that define the lowest and highest quartiles of PO_2_ and PCO_2_ were fairly consistent for all gestational age groups on all 3 postnatal days (Table 1). By and large, gestational age groups differed minimally in the boundaries for what defines an extreme quartile of lowest and highest PO_2 _and lowest and highest PCO_2_ quartiles. Invariably, measurements that define an extreme quartile tended to be most extreme on day 1.

Infants who had an abnormal blood gas measurement on multiple days were more likely than others to have had a high SNAPPE-II (Table 2). Newborns who had recurrent/persistent hypoxemia were more likely than their peers to have required 14 or more days of ventilation assistance. Those who had hypercapnea were more likely than others to have developed very severe BPD and showed a predilection to be having a birth weight that was low for gestational age, to be born to a woman who had fever during the pregnancy, and to have required 14 or more days of ventilation assistance.

The univariable risk profiles of severe and very severe BPD are similar (Table 3). Both are characterized by a tendency to include an overrepresentation of newborns in the lowest gestational age category, those born to a woman who experienced fever during this pregnancy, those with a high SNAPPE-II, and very high rates of ventilation on 14 or more days.

In multivariable analyses, hypoxemia was associated with both severe and very severe BPD, while hypocapnea was associated with a reduced risk of BPD not accompanied by a need for assisted ventilation (Table 4, top set). Hypercapnea however was associated with ventilator-dependent BPD. Adding a variable for 14 or more days of ventilation prominently reduced or eliminated the associations with hypoxemia and hypercapnea (Table 4, bottom set). On the other hand, doing so did not influence the association between hypocapnea and severe BPD and allowed the association between hypocapnea and very severe BPD to become statistically significant.

## 4. Discussion

In the present study, the first of its kind in the surfactant era, recurring/persistent blood gas abnormalities during the first three postnatal days convey information about the risk of severe and very severe BPD in ELGANs above and beyond the information conveyed by indicators of prematurity and risk of dying. The finding that some of the risk information is diminished when intervening ventilation is considered suggests that some of the blood gas disturbances are indicative of the need for ventilation, which in turn contributes to such late respiratory disorders as BPD.

In the ELGAN study sample, blood gas abnormalities on two of the first three postnatal days were associated with sustained or recurrent systemic inflammation in the week and a half that followed [22]. In addition, “prolonged” ventilation, defined as ventilation on 14 or more days between birth and 36 weeks PMA, was also associated with sustained or recurrent early systemic inflammation [29] and early sustained or recurrent systemic inflammation was associated with heightened BPD risk [30]. Consequently, we hypothesized that the blood gas derangements that predicted BPD probably contributed to BPD via an increased likelihood of “prolonged” ventilation, which, in turn, might promote inflammation [29]. The differences seen between the two sets of Table 4, which reflect addition of only the ventilation variable to multivariable models of BPD risks, indicate that ventilation is likely to be an intermediary between the blood gas derangements and the BPD.

In a previous study, components of SNAP-II contributed to the prediction of the need for CPAP and/or ventilator assistance 72 hours after birth among newborns whose gestational age was ≥34 weeks [31]. Thus, we consider the SNAPPE-II worthy of inclusion among variables adjusting for endogenous risk of BPD in ELGANs.

A previous study found an association between early hypercapnea and BPD [32], while another found an association between hypercapnea and BPD but only among the premature infants whose clinical course complicated by PDA [4]. We, too, found that hypercapnea was associated with very severe BPD, but not with severe BPD. The association with very severe BPD was diminished and lost its statistical significance when we added a variable for duration of ventilation. These findings are compatible with the view that the association of hypercapnea with very severe BPD reflects phenomena associated with prolonged ventilation, perhaps more than early gas adjustments.

The apparent protective effect of hypocapnea against BPD in the present study is in contrast to a report from the early surfactant era that hypocapnea before surfactant therapy was associated with an increased risk of BPD [15]. It is unclear how hypocapnea might protect against BPD.

We are reluctant to infer that exposure to the observed blood gas extremes contributed to BPD. Rather, we consider it highly probable that the blood gas extremes are the first indicators of the severity of the respiratory dysfunction that will result in severe and very severe BPD. Nevertheless, we are not yet prepared to dismiss the possibility that improved care to minimize the occurrence of some of the blood gas abnormalities might reduce the occurrence of severe BPD.

We minimized confounding by indication. To avoid attributing to hypocarbia what might more appropriately be attributed to its antecedents, one group of investigators created a hypocarbia propensity score when evaluating the presumed consequences of hypocarbia [33].

Among the variables that comprised the propensity score were low gestational age <26 weeks, low birth-weight *Z*-score, and ventilation. We included these in multivariable models of BPD risk. Other propensity score components, including labor, membrane rupture, maternal leukocytosis, and antenatal antibiotic treatment, are closely related to other variables we adjusted for (e.g., maternal fever during pregnancy and recovery of* Mycoplasma* from the placenta). Similarly, such hypocarbia propensity score components such as systemic hypotension on day 1, neonatal leukopenia on day 1, and administration of volume expanders and/or vasopressors on day 1, are closely related to high values of SNAPPE-II. Consequently, to a considerable extent we have approximated the hypocarbia-propensity score used previously. We acknowledge that our efforts might not have achieved the goal we set for our multivariable analyses.

Compared to infants exposed to pressure-limited ventilation equipment, those treated with volume-targeted ventilation have lower rates of hypocarbia, and the combined outcome of BPD/death [34, 35]. These types of findings appear to be contributing to a replacement of pressure-limited ventilation equipment with volume-targeted equipment. Such changes in ventilation might contribute to a reduced occurrence of BPD [36].

Our study is not without limitations. Our findings are based on* post hoc* analyses of data collected for a study of indicators of brain damage in ELGANs [19]. The boundaries for blood gas extremes were pooled values available for all ELGANs involved in this study who happened to have a wide range of respiratory illness severity. In addition, our definition of hypoxemia is not severe at all. Children, who died of their severe respiratory dysfunction before a BPD diagnosis could be made, are not included in the analyses. We did not have any specific index to evaluate the association between subsequent BPD and volutrauma/barotrauma. As with all observational studies, we are unable to distinguish between causation and association as explanations for what we found. Finally, even though we included SNAPPE-II scores in our multivariate regression models, we cannot completely rule out that the sickest infants were more likely to be treated aggressively than others who were not quite so sick, making our study prone to confounding by indication [37, 38].

Our study has several strengths. First, we included a large number of infants, making it unlikely that we missed important associations due to lack of statistical power, or claimed associations that might have reflected the instability of small numbers. Second, we selected infants based on gestational age, not birth weight, in order to minimize confounding due to factors related to fetal growth restriction [39]. This is especially important in light of the increased risk of BPD among infants in the ELGAN study who were born with severe growth restriction [30]. Third, we collected all of our data prospectively.

In conclusion, blood gas abnormalities in the first three postnatal days were associated with BPD, but adding a variable for duration of ventilation to the multivariable model left only hypocapnea associated with BPD. One reasonable implication of these findings is that hypoxemia and hypercapnea are probably not in the causal chain leading to BPD. Rather, they are likely indicators of the need for ventilation, which is more likely to contribute to BPD risk.

Another implication of our findings is that hypocapnea is also probably not in the causal chain. Future studies are recommended to identify why hypocapnea conveys information about the reduced risk of BPD.

## Figures and Tables

**Table 1:** The values that define the lowest and highest quartiles of each blood gas on each day in each gestational age group.

Blood gas quartile →	Lowest PO_2_ (mm Hg)			Highest PO_2_ (mm Hg)		
Postnatal day →	1	2	3	1	2	3

Gestational age (wks) ↓						
23-24	39	43	44	152	98	104
25-26	42	45	45	145	100	95
27	40	44	46	142	92	96

Blood gas quartile →	Lowest PCO_2_ (mm Hg)			Highest PCO_2_ (mm Hg)		
Postnatal day →	1	2	3	1	2	3

Gestational age (wks) ↓						
23-24	27	33	34	65	68	63
25-26	29	35.5	35	60	64	60
27	29	35	36	58	57	56

**Table 2:** The distribution of infants who had a blood gas extreme (defined as a P_a_O_2_ or PCO_2_ in the highest or lowest quartile for gestational age on at least two of the first three postnatal days) listed at the top of each column within strata of potential confounders, listed on the left. These are column percents.

Potential confounder	Blood gas extreme								
	Lowest		Highest		Lowest		Highest		Row
	P_a_O_2_		P_a_O_2_		PCO_2_		PCO_2_		
	Yes	No	Yes	No	Yes	No	Yes	No	*N*
Gestational age (weeks)									
23-24	23	25	24	25	25	24	26	24	223
25-26	47	46	44	47	46	46	44	47	418
27	30	29	32	29	28	30	30	29	265
Birth weight *Z*-score <−1^§^									
Yes	25	20	25	20	23	20	30	18	59
Maternal fever^†^									
Yes	8	6	7	6	7	7	10	5	209
Placenta *Mycoplasma *									
Yes	9	10	10	10	14	9	7	11	109
SNAPPE-II ≥45									
Yes	33	28	37	28	34	28	47	24	263
Days ventilated									
<7	14	22	19	21	21	20	6	25	186
7–13	9	11	8	11	9	10	10	10	92
14+	77	67	74	68	69	69	84	65	628
BPD									
None	9	9	8	9	9	9	7	9	80
Mild/moderate	30	36	33	35	42	32	25	37	314
Severe	47	45	48	45	39	48	50	44	474
Very severe	14	10	11	11	9	11	17	9	98
Maximum column *N*	195	711	189	717	201	705	197	709	906

^§^Birth weight *Z*-scores based on Yudkin et al. [14] standard.

^†^Maternal temperature >100.4°F during pregnancy.

**Table 3:** The distribution of infants who had the form of bronchopulmonary dysplasia listed at the top of each column within strata of potential confounders, listed on the left. These are column percents.

Potential confounder	Bronchopulmonary dysplasia				
	None	Mild/moderate	Severe	Very severe	Row
		O_2_	O_2_ − no vent	O_2_ + vent	
		at 28 weeks	at 36 weeks	at 36 weeks	*N*
Gestational age (weeks)					
23-24	0	17	33	34	223
25-26	30	50	45	52	418
27	70	33	22	14	265
Maternal fever^†^					
Yes	10	11	25	41	59
Birth weight *Z*-score <−1^§^					
Yes	6	**6**	**6**	**7**	209
Placenta *Mycoplasma *					
Yes	10	12	10	5	109
SNAPPE-II ≥45					
Yes	6	20	36	49	263
Days ventilated					
<7	66	32	8	0	186
7–13	20	16	6	2	92
14	14	52	86	98	628
P_a_O_2_ quartile on ≥2 days					
Lowest	23	18	22	29	195
Highest	19	20	22	21	189
PCO_2_ quartile on ≥2 days					
Lowest	23	27	19	19	201
Highest	18	16	24	35	197
Maximum column *N*	80	314	414	98	906

^§^Birth weight *Z*-scores based on Yudkin et al. [14] standard.

^†^Maternal temperature >100.4°F during pregnancy.

**Table:** (a) Without a variable for days of ventilation during the first 2 weeks (<7, 7–13, and ≥14)

BPD	Blood gas abnormalities			
	Low PO_2_	High PO_2_	Low PCO_2_	High PCO_2_
Severe	1.5 (1.02, 2.3)	0.9 (0.6, 1.4)	*0.6 (0.4, 0.96) *	1.3 (0.9, 2.0)
Very severe	2.5 (1.3, 5.0)	0.7 (0.3, 1.5)	0.5 (0.3, 1.03)	2.5 (1.2, 5.0)

**Table:** (b) With a variable for days of ventilation during the first 2 weeks (<7, 7–13, ≥14)

BPD	Blood gas abnormalities			
	Low PO_2_	High PO_2_	Low PCO_2_	High PCO_2_
Severe	1.2 (0.8, 1.9)	0.9 (0.6, 1.5)	*0.6 (0.4, 0.96) *	0.9 (0.6, 1.5)
Very severe	1.7 (0.8, 3.5)	0.8 (0.3, 1.8)	*0.4 (0.2, 0.9) *	1.9 (0.9, 4.2)

^§^All models are adjusted for conception assistance, maternal fever during pregnancy, birth weight *Z*-score <−1, recovery of a *Mycoplasma* from the placenta, and SNAPPE ≥45. These models also include a hospital group/cluster term to account for the possibility that infants born at a particular hospital are more like each other than infants born at other hospitals.
